# Mortality related to sickle cell disease and COVID-19 in Brazil, 2020

**DOI:** 10.1590/1806-9282.20231466

**Published:** 2024-05-13

**Authors:** Augusto Hasiak Santo

**Affiliations:** 1 Universidade de São Paulo (retired), Faculty of Public Health, Department of Epidemiology – São Paulo (SP), Brazil.

**Keywords:** Sickle cell disease, COVID-19, Multiple-cause-of-death, Mortality, Cause of death, Brazil

## Abstract

**OBJECTIVE:**

The ability to cause death is the definitive measure of an infectious disease severity, particularly one caused by a novel pathogen like severe acute respiratory syndrome-CoV-2 (COVID-19). This study describes sickle cell disease-related mortality issues during the COVID-19 pandemic in Brazil.

**METHODS:**

The provisional 2020 mortality data originated from the public databases of the Mortality Information System and were investigated using the multiple-cause-of-death methodology.

**RESULTS:**

In 2020, 688 sickle cell disease-related deaths occurred, of which 422 (61.3%) had an underlying cause of death and 266 (38.7%) had an associated cause of death. Furthermore, 98 COVID-19-related deaths occurred, of which 78 were underlying cause of death among sickle cell disease associated (non-underlying) cause of death. Sickle cell disease-related deaths occurred mostly among young adults aged 25–49 years. COVID-19 deaths occurred at ages older than among sickle cell disease-related deaths. Majority of deaths happened in the southeast (42.3%) and northeast regions (34.0%), while COVID-19 deaths prevailed in the northeast region (42.9%). Regarding overall deaths, the leading underlying cause of death was sickle cell disease itself, followed by infectious and parasitic diseases (14.8%), owing to COVID-19 deaths, and diseases of the circulatory system (8.9%). Next, in males, diseases of the digestive system (4.8%) occurred, while, in females, maternal deaths succeeded, included in the chapter on pregnancy, childbirth, and the puerperium, accounting for 5.9% of female deaths. The leading overall associated (non-underlying) cause of deaths were septicemias (29.4%), followed by respiratory failure (20.9%), pneumonias (18.3%), and renal failure (14.7%).

**CONCLUSION:**

In Brazil, COVID-19 deaths produced trend changes in sickle cell disease-related causes of death, age at death, and regional distribution of deaths in 2020.

## INTRODUCTION

The ability to cause death is the definitive measure of an infectious disease severity, particularly one caused by a novel pathogen like severe acute respiratory syndrome (SARS)-CoV-2 (COVID-19). Mortality rates improve the knowledge about the character of a disease, identify at-risk population, and evaluate the quality of health care^1^. Epidemiological findings suggest that persons with sickle cell disease (SCD) who are infected with COVID-19 have a higher risk of severe disease course and higher mortality rates compared with COVID-19 infections in the general population of similar ages^2^. The higher risk for serious illness from COVID-19 results from the chronic inflammation linked with enhanced risk of thrombosis, especially during a vaso-occlusive crisis. Patients with SCD are also immunocompromised due to spleen autoinfarction or surgical splenectomy often due to red-cell splenic sequestration. Furthermore, comorbidities and the cumulative impact of acute and chronic complications lead to progressive organ damage in SCD patients which increases the mortality risk from COVID-19-related SARS.

This paper aims to describe national mortality related to SCD during the COVID-19 pandemic in 2020 in Brazil using the methodology of multiple causes of death.

## METHODS

The provisional 2020 annual mortality data were extracted from the public multiple-cause-of-death databases of the Mortality Information System (Sistema de Informações Sobre Mortalidade – SIM) located at the Brazilian Unified Health System Information Technology Department (Departamento de Informática do Sistema Unico de Saúde – DATASUS), Ministry of Health (MH)^3^. All deaths were included in which SCD, three-character category D57 of the International Classification of Diseases and Related Health Problems, Tenth Revision (ICD-10), as a cause of death, was listed on any line or in either part of the WHO International Form of Medical Certificate of Cause-of-Death (the medical certification section of the death certificate), irrespective of whether they were characterized as the underlying cause of death (UCOD) or as an associated (non-underlying) cause of death (ACOD). Complications of the underlying cause (part I of the medical certification section) and contributing causes (part II of the medical certification section) were jointly designated as associated (non-underlying) causes of death^4-6^. We employed the 2020 mid-year estimates of the population for Brazil, discriminated by year, sex, age group, and Brazilian regions.

The World Health Organization (WHO), and the Ministry of Health, in Brazil, defined the guidelines to standardize the medical certification on ICD-10 mortality processing and UCOD selection for COVID-19-related deaths^7-9^. As a cause of death, COVID-19 should be identified using ICD-10 four-character subcategory B34.2 with pandemic markers U07.1 (COVID-19, virus identified) and U07.2 (COVID-19, virus not identified). The markers would help the investigation of the cause of death process, specifying if the diagnosis of COVID-19 was confirmed or not. Throughout the first semester of 2020, the code U04.9 was used to refer to SARS to identify deaths from COVID-19. In July 2020, a note or the MH revised the use of code U04.9 and addressed the use of code J98.8 instead or in conjunction^10^. The U04.9 code would only remain in the SIM if the SARS were part of the events chain that led to death.

Using mortality rates and proportions, we studied the distributions of the following variables: sex, age at death (in 5-year age groups), race/color, year of death, UCOD, associated (non-underlying) cause(s) of death, total mentions of each cause of death, mean number of causes listed per death certificate, and geographical distribution of deaths. SCD mortality rates (per one million population) as UCOD and ACOD for males and females were calculated. The expected number of deaths in 2020 was found by applying 2019 age-specific SCD-death rates to the 2020 population estimates. Standardized mortality ratios (SMRs) between 2019 and 2020 deaths were calculated. Medical and demographic variables were processed using the following software: dBASE III Plus, version 1.1, dBASE IV (Ashton-Tate Corporation, Torrance, CA), and Epi Info, version 6.04d (Centers for Disease Control and Prevention, Atlanta, GA), Excel 2016 (Microsoft Corporation, Redmond, WA). The Multiple Causes Tabulator (Tabulador de Causas Múltiplas for Windows) (TCMWIN, version 1.6) program (DATASUS, Ministério da Saúde, Faculdade de Saúde Pública, Universidade de São Paulo, Brazil) processed ICD-10 codes in the presentation of the associated causes and of the mean number of causes per death certificate^11^. The sex and age-adjusted crude and average mortality rates for the study period were standardized, by the direct method, to the new WHO Standard Population^12^. Crude and standardized rates were calculated according to the 5-year age groups.

We used analysis of variance to compare the mean number of causes mentioned on the death certificate and the Kruskal-Wallis H test to compare the mean age at death between groups. Statistical significance was set at p<0.05.

The study was waived by the author's institutional review boards because it exclusively uses large public domain national mortality databases of secondary data that are anonymous, without nominal personal identification, and therefore carries no individual risk whatsoever. The ethical principles contained in the Resolution of the National Health Council (CNS) n. 466 of December 12, 2012, were observed.

## RESULTS

The provisional data show 1,552,740 deaths recorded in Brazil during 2020. Of these, 688 were related to SCD, of which 422 (61.3%) identified as the UCOD and 266 (38.7%) as the ACOD (Table 1). Furthermore, among sickle cell-related deaths, 98 COVID-19-related deaths occurred as follows: 78 COVID deaths as UCOD along with 266 ACOD sickle cell; 6 deaths as ACOD cause with sickle cell UCOD; 6 deaths as ACOD with other UCOD causes, and 8 deaths coded as U04.9 with sickle cell as UCOD. In Brazil, all mentioned COVID-19 deaths occurred 14.2% among overall SCD-related deaths, of which 11.3% were the UCOD.

**Table 1:** Deaths related to sickle cell disease and COVID-19 according to the qualification of the cause of death, sex, age at death, race/color, and Brazilian regions, Brazil, 2020.

		SCD causes of death						COVID-19 total deaths		
		Underlying		Associated		Total				
		n	%	n	%	n	%	n	%	SPM%
All deaths		422	61.3	266	38.7	688	100.0	98	100.0	14.2
Sex										
	Male	211	50.0	122	45.9	333	48.4	48	49.0	14.4
	Female	211	50.0	144	54.1	355	51.6	50	51.0	14.1
Age at death (years)										
	00–04	20	62.9	4	37.1	24	3.5	2	2.0	8.3
	05–24	134	77.2	52	22.8	186	27.0	25	25.5	13.4
	25–49	177	73.5	129	26.5	306	44.5	46	46.9	15.0
	50–74	79	62.4	61	37.6	140	20.3	19	19.4	13.6
	75 and more	12	69.0	20	31.0	32	4.7	6	6.1	18.8
Race/color*										
	White	65	15.9	46	18.1	111	16.7	14	14.7	12.6
	Black	120	29.3	80	31.5	200	30.1	26	27.4	13.0
	Yellow	1	0.2	3	1.2	4	0.6	1	1.1	25.0
	Brown	221	53.9	125	49.2	346	52.1	54	56.8	15.6
	Indigenous	3	0.7	0	0.0	3	0.5	0	0.0	0
Brazilian regions										
	North	42	79.2	11	20.8	53	7.7	7	7.1	13.2
	Northeast	136	58.1	98	41.9	234	34.0	42	42.9	17.9
	Southeast	178	61.0	114	39.0	292	42.4	33	33.7	11.3
	South	20	57.1	15	42.9	35	5.1	4	4.1	11.4
	Center_West	46	62.2	28	37.8	74	10.8	12	12.2	16.2

Source:

Ministry of Health, Unified Health System Information Technology Department (provisional data). SPM% = Specific proportional mortality.

* Race/color available data on death certificates not totally complete.

**Figure 1:**
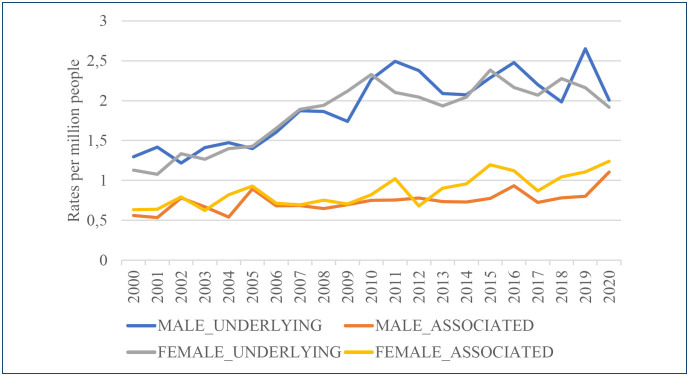
Age-standardized death rates related to sickle cell disease according to causes of death and sex in Brazil, 2000–2020.

Male and female 2020 standardized mortality rates were 2.02 and 1.92 per million for UCOD and 1.10 and 1.24 per million for ACOD (non-underlying), respectively. Figure 1 updates the mortality SCD-related trend since 2000, including 2019 and 2020 data. The SMRs resulting from the comparison between 2020 observed with expected SCD-related deaths, for males and females, as UCOD, were 0.76 and 0.89, and as ACOD, 1.45 and 1.18, respectively.

Around 82% of deaths occurred among brown and black persons. Majority of SCD-related deaths happened in the southeast region, mostly in São Paulo (18.5%), Rio de Janeiro (12.2%), and Minas Gerais (10.5%), followed by the northeast region, in the states of Bahia (18.6%). However, COVID-19-related deaths occurred in the northeast region, in Bahia (25.5%), and southeast, in Rio de Janeiro (16.3%) and São Paulo (13.3%). Otherwise, higher COVID-19-specific proportional mortality was observed in the northeast, center-west, and north regions. SCD-related deaths occurred mostly among young adults aged 25–49 years (Table 1).

The UCOD on 688 certificates for males and females in which SCD was listed as a cause of death is presented in Table 2, according to the ICD structure. SCD occurred in 61.3% of overall deaths. The second major UCOD was included in the ICD chapter on infection and parasitic diseases, tallying 14.8% of deaths, owing to 78 deaths caused by the coronavirus infection, followed by the circulatory system diseases. In males, gastrointestinal system diseases were observed, while, in females, maternal deaths, included in the chapter on pregnancy, childbirth, and the puerperium, accounted for 5.9% of female deaths.

**Table 2:** Underlying causes on death certificates that listed sickle cell disease as a cause of death, Brazil, 2020.

Underlying causes of death (ICD-10 chapters and rubrics)		Male		Female		Total	
		n	%	n	%	n	%
**Certain infectious and parasitic diseases (A00–B99)**		**52**	**15.6**	**50**	**14.1**	**102**	**14.8**
	Intestinal infectious disease (A00–A09)	1	0.3	4	1.1	5	0.7
	Tuberculosis (A15–A19, B90)	3	0.9	0	0.0	3	0.4
	Dengue and dengue hemorrhagic fever (A90–A91)	3	0.9	2	0.6	5	0.7
	Viral hepatitis (B15–B19)	4	1.2	2	0.6	6	0.9
	Human immunodeficiency virus (HIV) disease (B20–B24)	1	0.3	2	0.6	3	0.4
	Coronavirus infection, unspecified (B34.2)	39	11.7	39	11.0	78	11.3
**Neoplasm (C00–D48)**		**3**	**0.9**	**5**	**1.4**	**8**	**1.2**
**Diseases of blood and blood-forming organs (D50–D890)**		**215**	**64.6**	**217**	**61.1**	**432**	62.8
	Sickle-cell disorders (D57)	211	63.4	211	59.4	422	61.3
**Endocrine nutritional and metabolic diseases (E00–E90)**		**5**	**1.5**	**6**	**1.7**	**11**	**1.6**
	Diabetes mellitus (E10–E14)	3	0.9	5	1.4	8	1.2
**Diseases of the circulatory system (I00–I99)**		**30**	**9.0**	**31**	**8.7**	**61**	**8.9**
	Hypertensive diseases (I10–I13)	7	2.1	4	1.1	11	1.6
	Ischemic heart diseases (I20–I25)	1	0.3	2	0.6	3	0.4
	Pulmonary heart diseases and diseases of pulmonary circulation (I26–I28)	2	0.6	4	1.1	6	0.9
	Heart failure (I50–I51)	2	0.6	3	0.8	5	0.7
	Cerebrovascular diseases (I60–I69)	11	3.3	13	3.7	24	3.5
**Diseases of the respiratory system (J00–J99)**		**3**	**0.9**	**1**	**0.3**	**4**	**0.6**
**Diseases of the digestive system (K00–K93)**		**16**	**4.8**	**13**	**3.7**	**29**	**4.2**
	Disease of liver (K70–K76)	7	2.1	6	1.7	13	1.9
	Disorders of gall bladder, biliary tract, and pancreas (K80–K86)	4	1.2	2	0.6	6	0.9
	Other diseases of the digestive system (K90–K92)	1	0.3	2	0.6	3	0.4
**Diseases of the genitourinary system (N00–N99)**		**3**	**0.9**	**5**	**1.4**	**8**	**1.2**
	Renal failure (N17–N19)	2	0.6	4	1.1	6	0.9
**Pregnancy, childbirth, and the puerperium (O00–O99)**		**—**	**—**	**21**	**5.9**	**21**	**3.1**
**Other underlying causes of death**		**6**	**1.8**	**6**	**1.7**	**12**	**1.7**
**Total**		**333**	**100.0**	**355**	**100.0**	**688**	**100.0**

Source:

Ministry of Health, Unified Health System Information Technology Department Rubrics and codes of the International Statistical Classification of Diseases and Related Health Problems, Tenth Revision. Bold values refer to the titles of ICD-10 Chapters, while the indented causes of death refer to its included rubrics.

The leading associated (non-underlying) causes of death listed on UCOD death certificates with SCD and other selected conditions for 2020 include septicemias, occurring in 29.4% of overall deaths, followed, among sickle cell UCOD, by pneumonia, respiratory failure, and renal failure, whereas, among other UCODs, by respiratory failure, shock, renal failure, pneumonia, and hypertensive diseases. The main ACODs related to COVID-19 deaths were respiratory failure (33.7%), SARS (26.5%), pneumonia (26.5%), septicemias (23.5%), and renal failure (12.2%).

## DISCUSSION

This study describes the mortality related to SCD in Brazil in 2020 looking for the impact of the COVID-19 pandemic on its course and characteristics by means of the comparison with 2000–2018 and 2019 data. As in the previous study, the multiple-cause-of-death methodology was used to profit from its benefits considering all mentioned death certificates where SCD was listed as a cause of death^13^. Note that the Ministry of Health emphasizes the use of multiple causes as mandatory to the study of the COVID-19 mortality^8^.

Overall, in 2020, SCD was identified as the UCOD in 61.3% of death certificates. This proportion is the lowest ever observed since the start of multiple-cause methodology use in Brazil. The average percentage of SCD as UCOD from 2000 to 2018 was 70.5%, and in 2019, it reached 71.6% among 714 SCD-related deaths. Therefore, SCD deaths were relocated as ACOD (non-underlying), reaching 38.7% among all mentioned ones. SCD ACOD attained then the highest value in the historical series, greater than 28.4% verified among 2019 related-SCD deaths. COVID-19 deaths, 29.3% among ACOD, are accountable for this result.

The description of SCD mortality trends was presented before^13^. The highest UCOD mortality rates were verified for females in 2015, followed by leveling, and for males in 2019, dropping in 2020 (Figure 1). Owing to COVID-19 deaths, the highest ACOD mortality rates occurred in 2020. Compared with the 2019 deaths, the SMRs confirmed the drop in UCOD and the increase in ACOD. The investigation of SCD-related mortality in the United States in 2020, compared with previous years, also identified an increase in deaths during the pandemic considering all mentions of SCD-related mortality^14^.

The distribution of deaths among Brazilian regions in 2020 in comparison with 2000–2018 and 2019 deaths shows less non-significant proportional increases in the north, northeast, and south regions and a decrease in the southeast region. Otherwise, the predominance of COVID-19 deaths in the northeast region, in the state of Bahia, is a subject of concern and further studies. The state of Bahia concentrates the largest proportion of hemoglobinopathies, particularly SCD, in Brazil^15^. Additionally, a survey of COVID-19 deaths in Belo Horizonte, Salvador, and Natal in 2020 observed that Salvador concentrated 57.7% of COVID-19 deaths and whose proportion in the total number of deaths was much higher than in the other two capitals^9^, such as also observed among SCD-related deaths.

The distinct outline of UCOD distribution among SCD-related mortality during 2020, in comparison with former years, points to the severity of COVID-19 deaths. Secondary only to SCD as the most frequent specific UCOD, COVID-19 deaths displaced even the cerebrovascular diseases and, considering ICD-10 chapters, the infectious and parasitic diseases replaced the circulatory system diseases. Among female deaths, the increase in the maternal proportional mortality of 5.9% in comparison with the 4.6% average verified in 2000–2018^14^ must be emphasized owing to three COVID-19 as ACOD.

While in Brazil, COVID-19 deaths occurred 14.2% with SCD-related deaths, of which 11.3% were identified as the UCOD, in the United States, COVID-19 deaths were associated with 8.4% of SCD-related deaths^14^. To the best of our knowledge, the United States and the Brazilian are the only ones available published country mortality studies over this time.

The overall outline of ACOD (non-underlying causes) studied in 2020 follows an equivalent composition as in the 2000–2018 period when related to SCD UCOD or other UCOD^13^. Septicemias, respiratory failure, pneumonia, renal failure, shock, hypertensive, and cerebrovascular diseases stand for the most frequently associated causes. Identified as an ad hoc cause of death owing to the COVID-19 pandemic, SARS is mentioned as ACOD among others UCOD, split according to the proposed codes (U04.9 and J98.8), following previous paper approach^9^.

This study has many limitations. Without doubt, the provisional mortality data for 2020 is the main question. COVID-19 is a new epidemic disease and its record may be hampered by the lack of knowledge by the medical community. Otherwise, some deaths may be under revision by the surveillance system until their inclusion or removal from mortality data. Otherwise, the official guidance on COVID-19 coding as a cause of death occurred in May 2020; as before the U04.9 was used to identify SARS meant for COVID-19 during the first semester of 2020. Notice that, in this paper, deaths including U04.9 are identified as ACOD and remain with their original UCOD. Therefore, despite the severity of the pandemic, COVID-19 deaths were underreported as a cause of death in the first semester of 2020. Conversely, the crude mean number of 3.67 causes per overall death certificate and the unprecedented crude mean number of 5.02 per COVID-19 UCOD certificates stand for the quality of death certification of SCD-and COVID-19-related deaths, as referred elsewhere^9^. The COVID-19 mean number results from its double codification as B34.2 with its mark numbers U07.1 or U07.2.

## CONCLUSION

In Brazil, the COVID-19 pandemic produced outlined changes in SCD-related causes of death, age at death, and regional distribution of deaths in 2020. SCDs as UCOD were relocated to ACOD due to the severity of COVID-10. The prevalence of COVID-19 deaths in the northeast region, especially in the city of Salvador, deserves further research, as well as the infectious and parasitic diseases as the second most frequent UCOD and the proportional rise of maternal deaths in females.
